# Domain-Based Functional Improvements in Bipolar Disorder After Interpersonal and Social Rhythm Therapy

**DOI:** 10.3389/fpsyt.2022.767629

**Published:** 2022-02-14

**Authors:** William Moot, Marie Crowe, Maree Inder, Kate Eggleston, Christopher Frampton, Richard J. Porter

**Affiliations:** Department of Psychological Medicine, University of Otago, Christchurch, New Zealand

**Keywords:** bipolar disorder, psychotherapy, domain-based, function, functional improvements, IPSRT

## Abstract

**Background:**

Studies typically report overall change in function when assessing bipolar disorder (BD) interventions, but individual domains are not analyzed. Which aspects of functioning are impacted is clearly important and may differ between treatments.

**Methods:**

Data were analyzed from two previous clinical trials of Interpersonal and Social Rhythm Therapy (IPSRT) for BD patients. Change in total and subscale scores on the Social Adjustment Scale Self-Report (SAS-SR) from 0 to 78 weeks, were analyzed.

**Results:**

152 BD patients took part in randomized controlled trials of IPSRT (*n* = 38) vs. Specialist Supportive Care (SSC) (*n* = 43), and of IPSRT (*n* = 41) vs. treatment as usual (TAU) which was discharge to primary care (*n* = 30). IPSRT was superior to TAU on change in the social and leisure activities and extended family subscales, and SAS-SR total score over 18 months.

**Limitations:**

Studies were not designed to be pooled. Patients in study 1 were younger and symptomatic at baseline. Patients assigned to TAU were more likely to drop-out. Patients did not respond to subscales that were not personally applicable (work, marital, children).

**Conclusion:**

IPSRT had a positive impact on two SAS-SR subscales compared to TAU over 18 months. Other subscales were limited by the lack of respondents due to individual applicability. Different psychotherapy may have differential effects on different domains of function. Measures of function and research into functioning in BD should include domain-based measures, and report the numbers of participants who respond to questions in each domain.

## Introduction

Patients with symptoms of bipolar disorder (BD) are more likely to experience impaired occupational (1), relational (2), and cognitive functioning (3). Furthermore, functional impairment can persist even when symptomatic remission has been achieved (4). A review of 17 studies of psychosocial outcomes in BD found 30–60% of patients experience functional impairment even during remission of symptomatic episodes (5). For many patients with BD, functional improvement is more important than symptomatic outcomes (6). Recognizing this, the traditional clinical emphasis on acute symptom reduction in BD has shifted to include longer-term focus on recovery of functioning in everyday life (7).

An important issue is how to consistently and accurately measure function. A recent review indicates ambiguity in the definition and measurement of functioning in BD (4). Researcher's personal definitions of functioning (8), and ability to interpret findings (9) may contribute to their choice of measure. There is no clear consensus on the most appropriate measure, despite a wide variety having been developed. Currently, the majority of studies use clinician-rated measures with far fewer including a self-report measure (4).

One self-report measure is the Social Adjustment Scale Self-Report (SAS-SR) (10). The SAS-SR is a measure of social functioning that was developed by adapting existing scales that had demonstrated sensitivity and utility in assessing role impairment. Development was driven by increased interest in social adjustment as opposed to symptomatology. The SAS-SR contains 45 questions (updated from the initial 42) that measure six major areas of functioning: work (as a paid worker, home-maker, or student); social and leisure activities; relationships with extended family; marital role; parental role; and role within the family unit. Each question is rated on a five-point Likert scale with a higher score indicating impairment (patients may leave questions blank if they are not applicable). Early and ongoing independent research found a high level of consistency between patient self-report and clinician assessment of patient function (11–13). Studies using the SAS-SR in BD samples have found significant baseline impairment in work-related performance, social and leisure activities, and family unit interactions compared with psychologically healthy population samples (14, 15).

Studies of psychotherapy for BD such as Interpersonal and Social Rhythm Therapy (IPSRT) have often examined effects of treatment on global function. Few have, however, examined the effects on individual domains. For example, Hoberg et al. (16) found a significant improvement in BD patient function from baseline to 12 weeks after 2 weeks of intensive group IPSRT [using the Sheehan Disability Scale (SDS)], but did not examine individual domains. Hlastala et al. (17) found significant improvements in overall function on the children's Global Assessment Scale (C-GAS) in a group of BD adolescents undergoing modified IPSRT for adolescents over 20 weeks and Steardo et al. (18) found BD patients had global improvements in function on the Global Assessment of Functioning (GAF) over 12 weeks of IPSRT. Frank et al. (19) however, examined functioning in a particular domain and found that BD patients receiving IPSRT showed a rapid initial improvement in occupational functioning (measured on the UCLA Social Attainment Scale) compared with those assigned to Intensive Clinical Management (ICM). However, this difference was not sustained after 2-years of follow-up.

Domain-specific assessment provides additional information regarding functioning, as it is likely that individual patients experience impairment in different domains. For instance, a recent systematic review and meta-analysis demonstrated that a higher proportion of BD patients experience impairment in occupational functioning (65.6%) than global functioning (58.6%) or other domains (20).Residual symptoms appear to have negative effects on some domains of functioning but not others (21). Additionally, while the GAF is the most commonly used global functioning measure in BD research (4), service users rate it as inappropriate and poorly relevant (22). Clinically, identifying which domains of functioning are most impaired and providing treatment that is aimed at improving these areas is of importance. Therefore, understanding which domains of functioning are improved by current treatment is highly relevant.

We used the SAS-SR as the primary measure of functioning in two previous Randomized Control Trials (RCTs) examining the efficacy of Interpersonal and Social Rhythm Therapy (IPSRT) compared with Specialist Supportive Care (SSC) (23) and IPSRT compared with treatment as usual (TAU) (24). Both studies found significant improvements in social functioning as measured by mean SAS-SR total score when undergoing psychotherapy (IPSRT or SSC), whilst patients randomized to TAU did not improve. In the second study, there was a significantly greater improvement during treatment with IPSRT compared with TAU. In this *post-hoc* analysis we have pooled the data from both RCTs, providing the opportunity to examine functional outcomes in a larger number of patients receiving psychotherapy for BD. Here we report a secondary analysis of these studies, examining the effects of IPSRT on domains within the SAS-SR and comparing these effects with SSC and TAU. We hypothesized that there may be greater changes in particular areas of function related to interpersonal relationships, such as extended family, family unit and marital domains, as a result of IPSRT.

## Methods

Data are from two randomized control trials (RCTs) of IPSRT for BD referred to as study 1 (23) and study 2 (24). All patients who participated in 18 months of structured therapy or TAU during these trials were considered eligible for *post-hoc* combined analysis.

### Inclusion/Exclusion Criteria

In study 1, patients were aged 15–36 years with BD-I, BD-II or BD not otherwise specified (defined as fulfilling the criteria for BD-II, with 2 days of hypomania). There were no criteria regarding mood state at entry. In study 2, patients had a diagnosis of BD-I or BD-II, were aged 18–64 years and did not meet the criteria for an episode of depression, mania, or mixed state at baseline. Exclusion criteria for both studies were minimal and included a primary diagnosis of schizophrenia, schizoaffective disorder, or severe substance use disorder (SUD).

### Assessment

The Structured Clinical Interview for DSM–IV Axis I Disorders (SCID-I) (25) and for Axis II Disorders (SCID-II) (26) were used to confirm psychiatric diagnoses. The cumulative burden of mood symptoms was measured using the Longitudinal Interval Follow-up Examination (LIFE). The LIFE is used to retrospectively rate the severity of depression and mania on a weekly basis over the previous 6 months (27). Ratings were carried out by a trained research assistant, by telephone, blind to treatment. In both studies, mood was also rated at baseline using the Young Mania Rating Scale (YMRS) (28).

Patients completed the SAS-SR, a 45-item self-report questionnaire measuring patient function over the previous 2 weeks. A score is derived from 7 subscale scores, which are averaged to give a final score in the range of 1–5, with a lower score reflecting greater social adjustment (10). In order that they appear on the SAS-SR the subscales are: How things have been going at work (work), how household tasks have been going (housework), how relationships with friends have been going and how spare time has been spent (social and leisure activities), how relationships with family excluding partners or children living at home have been going (extended family), how things have been going with a partner who lives with you (marital), how things have been going with children living at home (children) and how things have been with immediate family living at home (family unit). We examined change in SAS-SR subscale scores between baseline and 78 weeks.

### Psychotherapeutic Intervention

In study 1 (23) patients were randomized to receive IPSRT or Specialist Supportive Care (SSC). In study 2 (24) patients were randomized to IPSRT or Treatment as Usual (TAU).

In both studies IPSRT was delivered according to a manualized protocol. IPSRT combines Interpersonal Psychotherapy with Social Rhythm Therapy to help patients reduce stressors that lead to relapse and to learn to live with bipolar disorder and its impact on their lives (29). The timing of sessions was flexible based on clinical need, usually consisting of 10–12 weekly sessions, followed by 6–8 fortnightly sessions, and 4–5 monthly sessions thereafter, with a total of ~24 sessions.

SSC was designed as a control psychotherapy based on American Psychiatric Association (APA) guidelines for the management of BD (30). SSC combines supportive psychotherapy and psychoeducation, with the focus of each session initiated by the patient. It is not organized around a systematic exploration of interpersonal issues or social rhythms.

Patients assigned to TAU remained under usual care from their general practitioner and were provided with information about education and services by Bipolar Support Canterbury.

For all psychotherapy patients, treating psychiatrists made medication changes using clinical judgment and guided by a decision tree to optimize psychopharmacological treatment. Medication decisions were consistent with the APA (30) and Royal Australian and New Zealand College of Psychiatrists (RANZCP) Guidelines (7) for the treatment of BD.

### Ethics

Both studies gained ethical approval from the Canterbury Ethics Committee (study 1) and New Zealand Health and Disability Commission (study 2). They were registered prospectively with the Australia and New Zealand Clinical Trials Registry (study 1—ACTRN12605000722695; study 2—ACTRN12611000961943).

### Primary Outcome Measures

In study 1 the primary outcome was the cumulative burden of depressive symptoms as measured by the LIFE. Study 2 had two primary outcomes: time to relapse and readmission to hospital. In this pooled analysis, outcome measures determined a priori were changes in function as measured by SAS-SR subscale scores.

### Statistical Analyses

Analyses used the Statistical Package for the Social Sciences (SPSS) version 25. Baseline demographic and clinical characteristics of patients in the four treatment arms were recorded using means, standard deviations, counts and frequencies where appropriate. Between group differences were examined using Fisher's protected least significant difference test for continuous variables, and *post-hoc* Chi-square tests for categorical variables.

The primary analysis used a univariate general linear model (GLM). For this analysis, patients randomized to IPSRT in study 1 and study 2 were grouped, as the primary goal was to examine a pooled group of participants receiving 18 months of IPSRT. These Dependent variables were SAS-SR subscale score changes from 0 to 78 weeks (work, housework, social and leisure activities, extended family, marital, children and family unit). Data distribution was then assessed with Kolmogorov-Smirnov and Shapiro-Wilk Tests of Normality. Pearson's correlations for parametric continuous variables, Spearman's correlations for non-parametric continuous variables, and One-Way ANOVA for categorical variables were used to test for significant correlations of sample characteristics with SAS-SR subscale score change. Variables identified as significantly correlated with each subscale score change were then entered in the GLM as co-variates. Work was co-varied for BD Type (BDI/BDII/BD-NOS). Housework was co-varied for Gender and BD Type (BDI/BDII/BD-NOS). Marital was co-varied for the presence of rapid-cycling. Children was co-varied for Gender. All dependent change variables were co-varied for their corresponding score at baseline. Each GLM was also co-varied by age, age of onset of first affective episode, baseline LIFE score, history of lifetime anxiety disorder, and mood state at baseline (not in episode/manic/hypomanic/depressive) to control for the significant differences between treatment arms at baseline. Randomization was entered as a fixed factor (IPSRT study 1 + study 2/ SSC study 1/ TAU study 2).

A secondary analysis of the IPSRT groups (IPSRT study 1 + IPSRT study 2) was performed, using a one sample *t*-test for each SAS-SR subscale score change. SSC and TAU were excluded, as the intention of this secondary analysis was to examine whether IPSRT had a significantly positive impact on each of the SAS-SR subscale scores.

## Results

### Sample Characteristics

In study 1, 100 patients were randomized to IPSRT (*n* = 49) or SSC (*n* = 51). Eighty-one patients completed the study, 38 (78%) and 43 (84%) in each respective arm. In study 2, 88 patients were randomly assigned to IPSRT (*n* = 43) or TAU (*n* = 45). Seventy-one patients completed the study, 41 (95%) and 30 (67%) in each respective arm. Therefore, 152 patients were included in the analyses.

Baseline demographic and clinical characteristics of all patients grouped by treatment arms are presented in Table 1. Given study 1 specifically recruited younger patients (aged 15–35), as expected there was a difference (*p* < 0.001) in age between the study groups. Differences were also demonstrated in lifetime rates of Anxiety Disorders (*p* < 0.05) and LIFE score at baseline (*p* < 0.05) between treatment arms. Age of onset was later in the TAU group compared with the other treatment arms (*p* < 0.05). In study 2, patients were specifically recruited out of episode resulting in a less symptomatic population at baseline.

**Table 1:** Clinical characteristics by treatment randomization.

	**IPSRT 1**		**SSC**		**IPSRT 2**		**TAU**	
	**(*N* = 38)**		**(*N* = 43)**		**(*N* = 41)**		**(*N* = 30)**	
**Characteristic**	***N***	**%**	***N***	**%**	***N***	**%**	***N***	**%**
Age (M ± SD)	27.3 ± 6.1^a^		26.8 ± 5.8^a^		40.8 ± 14.0^b^		42.2 ± 12.8^b^	
Gender (F)	29	76	33	77	31	76	23	77
Ethnicity (Pākehā)	33	87	34	79	31	76	22	73
Bipolar 1/2	30/4	79/11	35/8	81/19	28/13	68/32	23/7	77/23
Index episode (depressive)	33	84	41	93	33	83	23	79
Rapid cycling	13	34	13	30	7	17	3	10
Age at onset (M ± SD)	16.7 ± 5.1^A^		14.9 ± 5.6^A^		17.3 ± 6.9^A^		20.7 ± 9.9^B^	
Lifetime anxiety disorder	19^A, B^	50	26^B^	60	11^A^	27	7^A^	23
Lifetime substance use disorder	18	47	23	53	14	34	11	37
Medication use^†^								
Lithium	13	34	13	30	12	29	11	37
Anticonvulsant mood stabilizer	14	37	17	40	17	41	6	20
Antipsychotic	19	50	22	51	21	51	21	70
Antidepressant	21	55	20	47	23	56	16	53
Drop out	11^A, B^	22	7^A, B^	14	3^B^	7	16^A^	36
SAS total score^†^ (M ± SD)	2.3 ± 0.5		2.3 ± 0.5		2.1 ± 0.4		2.1 ± 0.5	
Cumulative mood score (LIFE)^‡^ (M ± SD)	2.1 ± 1.1^A^		1.8 ± 1.4^A^		1.1 ± 1.2^B^		0.8 ± 1.2^B^	
YMRS^†^ (M ± SD)	1.7 ± 3.9		2.5 ± 3.0		1.5 ± 2.9		2.3 ± 3.1	

† *At 0 weeks.*

‡ *Retrospective from 0 to 26 weeks.*

*^a, b^Rows with differing superscript letters denote a significant difference at the p < 0.001 level.*

*^A, B^Rows with differing superscript letters denote a significant difference at the at p < 0.05 level*.

Patients were significantly more likely to drop out of the study if they were randomized to TAU. Analysis of the drop-out group's baseline demographics and clinical characteristics showed similar characteristics as patients were more likely to be older (*p* < 0.05) and older at age of onset of any affective episode (*p* < 0.05) in TAU than other treatment randomization. There were no significant differences found in mood-related measures and no significant differences found between dropouts and completers.

### Outcomes

**Figure 1:**
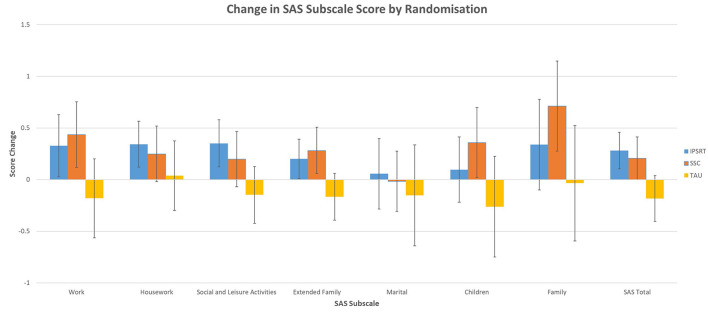
Treatment randomization on SAS subscale score change—Estimated Marginal Means (EMM) with 95% Confidence Intervals (CIs) from GLM.

The primary analysis was of the effect of treatment randomization on change in SAS-SR subscale score from 0 to 78 weeks (see Figure 1).

Initial GLM results (see Table 2) showed a significant association of treatment randomization on the social and leisure activities subscale (*p* = 0.030), extended family subscale (*p* = 0.018) and SAS-SR total score (*p* = 0.011). Sub analysis using pairwise comparisons (see Table 3) showed IPSRT had a significant effect compared with TAU on the social and leisure activities subscale (*p* = 0.009), extended family subscale (*p* = 0.020) and SAS-SR total score (*p* = 0.002). There was no difference between IPSRT and SSC. There were significantly fewer degrees of freedom in the work, marital, children and family unit subscales due to the lower number of people who completed these sections.

**Table 2:** Treatment randomization on SAS subscale score change—significant predictive values from GLM.

		**95% confidence interval of the difference**				
	**EMM^†^**	**Lower**	**Upper**	**df**	***F***	***p***
	**(IPSRT/SSC/TAU)**	**(IPSRT/SSC/TAU)**	**(IPSRT/SSC/TAU)**			
Work (*n* = 74)	0.329/0.436/−0.181	0.028/0.119/−0.564	0.629/0.753/0.202	2, 49	1.424	0.250
Housework (*n* = 149)	0.343/0.249/0.038	0.122/−0.021/−0.298	0.564/0.519/0.374	2, 101	0.067	0.856
Social and leisure activities (*n* = 152)	0.351/0.197/−0.148	0.123/−0.071/−0.422	0.578/0.465/0.126	2, 130	3.598	0.030
Extended family (*n* = 150)	0.200/0.282/−0.166	0.009/0.056/−0.393	0.390/0.507/0.061	2, 128	4.165	0.018
Marital (*n* = 58)	0.056/−0.017/−0.152	−0.286/−0.307/−0.640	0.398/0.274/0.337	2, 32	0.521	0.599
Children (*n* = 54)	0.097/0.359/−0.262	−0.219/0.021/−0.749	0.413/0.697/0.225	2, 30	2.084	0.142
Family (*n* = 74)	0.339/0.712/−0.035	−0.101/0.274/−0.595	0.779/1.149/0.524	2, 54	2.054	0.138
SAS total (*n* = 152)	0.280/0.206/−0.183	0.103/−0.002/−0.405	0.457/0.414/0.038	2, 130	4.712	0.011

† *Estimated Marginal Means*.

**Table 3:** Secondary pairwise comparison analysis of IPSRT with SSC and TAU based on significant predictive values from GLM.

	**Pairwise comparison with SSC**		**Pairwise comparison with TAU**	
	**Dif. ±SE^†^**	***p***	**Dif. ±SE^†^**	***p***
Social and leisure activities	0.154 ± 0.173	0.375	0.499 ± 0.188	0.009
Extended family	−0.082 ± 0.147	0.577	0.365 ± 0.155	0.020
SAS total	0.078 ± 0.133	0.559	0.465 ± 0.144	0.002

† *Difference between Estimated Marginal Means ± Standard Error, as estimated by GLM*.

Secondary analysis examined the effect of IPSRT from baseline to 18 months on SAS-SR subscale score change for all participants (see Table 4). Housework (*p* = 0.012) and social and leisure activities (*p* < 0.001) showed statistically significant mean score improvements of 0.249 and 0.374 respectively.

**Table 4:** SAS subscale score change from baseline for IPSRT.

**One sample *t*-test**					
		**95% confidence interval of**			
		**the difference**			
**SAS subscale score change**	**Mean difference**	**Lower**	**Upper**	***t***	***p***
Work (*n* = 38)	0.097	−0.196	0.389	0.668	0.509
Housework (*n* = 78)	0.249	0.056	0.442	2.564	0.012
Social and leisure activities (*n* = 79)	0.374	0.209	0.548	4.517	<0.001
Extended family (*n* = 78)	0.094	−0.058	0.246	1.237	0.220
Marital (*n* = 22)	0.171	−0.038	0.379	1.703	0.103
Children (*n* = 28)	0.259	−0.075	0.593	1.590	0.123
Family (*n* = 38)	0.149	−0.184	0.482	0.908	0.370
SAS total (*n* = 79)	0.318	0.181	0.456	4.612	<0.001

After completing these analyses, power calculations were performed on each SAS subscale change and SAS Total Score change. Table 5 shows that sufficient power to detect an effect of IPSRT over TAU was present in the Social and Leisure Activities, Extended Family and SAS Total Score change scales.

**Table 5:** Power calculations for SAS total and subscales—IPSRT compared with TAU.

**Power calculations**			
**SAS subscale score change**	**Observed effect size**	**Observed differences**	**Detectable differences (80% power) with the observed Ns**
Work	0.820	0.509	0.54
Housework	0.467	0.297	0.39
Social and leisure activities	0.818	0.453	0.34
Extended family	0.663	0.35	0.34
Marital	0.456	0.244	0.53
Children	0.591	0.312	0.56
Family	0.571	0.472	0.76
SAS total	0.995	0.438	0.27

## Discussion

In this pooled analysis of two RCTs examining psychotherapy in BD, there was a significant effect of treatment randomization on the social and leisure activities SAS-SR subscale and extended family SAS-SR subscale. Effect of treatment randomization was also found on SAS-SR Total score, confirming findings from our previous analyses (23, 24). Further examination of adjusted score change in the different therapy arms suggested that this is driven by a greater effect of IPSRT in these domains, with *post-hoc* analysis showing a significant difference between IPSRT and TAU on the social and leisure activities subscale and extended family subscale. No significant differences were found on the other subscales. No significant differences were found between IPSRT and SSC. It is important to note that housework, social and leisure activities, and extended family subscales had the greatest number of respondents, close to the total number of respondents at follow-up. In contrast, work, marital, children, and family unit subscales each had less than half the number of respondents at baseline and follow-up, which will have contributed to the lack of statistically significant findings in these domains.

Examination of baseline scores compared with follow up scores in the IPSRT groups showed significant improvement from baseline to 18 months in the housework and social and leisure activities SAS-SR subscales, and SAS-SR total score. The improvements seen in the social and leisure activities and extended family subscales may be attributable to the enhanced interpersonal skills, promoted by IPSRT. The establishment of circadian stability is promoted by the Social Rhythm component of IPSRT and the therapeutic mechanisms of the IPT component are related to decreasing interpersonal stress, facilitating emotional processing, improving interpersonal skills and enhancing social support (31). It is interesting to note, however, that other Interpersonal SAS-SR subscales (marital, children and family unit) did not see significant improvements with IPSRT compared with TAU which involved no psychotherapy. There were significantly fewer respondents in several of the SAS-SR subscales (work, marital, children and family unit) (see Table 2). This is likely due to the design of the SAS-SR, where participants do not respond to items that are not relevant to them (e.g., non-response to marital subscale if single).

Previous studies using the SAS-SR have detailed only results of overall score change, and used this as a proxy for improvement in social functioning. It is of interest that many of our patients did not answer questions across several domains, due to the lack of individual applicability. The SAS-SR is scored by averaging scores across all the questions answered. These results emphasize the fact that what is measured and reflected in this score varies across individuals depending on which domains are applicable. Individuals may not be working, married, or have children and therefore are unable to answer questions on these subscales. These scores are then unlikely to change over the course of treatment, limiting the ability of the SAS-SR to demonstrate improvement in functioning in these areas over time.

Two previous studies employed a different measure, the Functional Assessment Short Test (FAST), to assess domain-specific functional improvements after intervention (24, 25). The FAST scale is (in contrast to the SAS-SR) a quick, clinician-rated measure comprising 24 items divided into six domains of function; autonomy, occupational functioning, cognitive functioning, financial issues, interpersonal relationships and leisure time with a higher score denoting a poorer outcome (32). The FAST scale does not have any questions that may not be answered due to applicability, for example in the occupational functioning section, patients who do not have a job are assigned a 3: the highest possible score. Rosa et al. (24) examined results over 6 months of engagement in the Bipolar Disorders Program of the Hospital Clinic at the University of Barcelona, which included pharmacotherapy, biophysical therapies such as electroconvulsive therapy, and psychoeducation as appropriate. They found improvements in autonomy, cognitive functioning, and interpersonal relationships at 21 days, with improvements in the work subscale at 3 months, and financial and leisure subscale improvements at 6 months. Torrent et al. (25) found improvements in only 2 of the 6 domains assessed by the FAST scale (interpersonal and occupational) when comparing 21 weeks of a weekly functional remediation program (intervention addressing neurocognitive issues with focus on enhancing function in daily routine) with TAU.

Miklowitz et al. (33) employed the Longitudinal Interval Follow-Up Evaluation–Range of Impaired Functioning Tool (LIFE-RIFT) to compare domain-specific functional improvements between 30 one-hour sessions (21 weekly and nine biweekly) of intensive psychosocial treatment (IPSRT, Family-Focused Therapy, or Cognitive Behavioral Therapy) and collaborative care (a 3-session psychoeducational treatment). The LIFE-RIFT is a quick, clinician-rated tool of nine items divided into four domains: relationships (family, children, or friends), satisfaction (contentment and fulfillment from activities with family and friends, job, and finances), work/role performance (employment, household, or student roles), and recreational activities/hobbies (34). Patients were recruited from the Systematic Treatment Enhancement Program for Bipolar Disorder (STEP-BD) (32) who were able to respond to all questions on the LIFE-RIFT. They found improvements in 2 of 4 domains: relationships and satisfaction, when comparing IPSRT alone and intensive psychotherapy as a whole, with collaborative care. Our study falls broadly in line with these studies, in that there were improvements on the social and leisure activities and extended family subscales when compared to treatment as usual. Both Rosa et al. and Torrent et al. identified improvements in interpersonal skills and associated this with exercises completed as part of the assigned therapy. Miklowitz et al. (33) postulated that changes in life conditions, self-esteem, mood, and functioning often occur among bipolar disorder patients undergoing intensive treatments, although these changes do not necessarily occur at the same time or as a direct result of each other.

The discrepancies between our own findings and results published by Rosa et al., Torrent et al., and Miklowitz et al. may be attributable to the differences in functional measurements and therapies implemented. Clinician-based measures inherently bias understanding of patient functioning, as the perception of the relationship between symptoms and psychosocial functioning differs significantly between clinicians and patients (35). We used a self-report measure which may have aided in avoiding sources of clinician bias. Both studies (36, 37) using the FAST scale did not have any difficulties with non-respondents, as the FAST scale relies on generally applicable questions. Miklowitz et al. (33) had a very large BD population to sample patients from, and recruited specifically for patients that were able to answer every question on the LIFE-RIFT. This further highlights the difficulty in accurately assessing patient function, as typically not all domains of function will apply to every patient. Assessment of occupational function has proven to be particularly problematic—for example the SAS-SR does not account for job loss or gain over the study period, which would impact statistical and individual results. Similarly, the FAST assigns a score of 3 to patients who are unemployed, however it does not assess whether unemployed patients have the capacity to engage in work, and subsequent work gainers of this nature could be transitioned from a poor score of 3 to the top score of 0 by virtue of gaining employment. It is unclear what the optimal method of assessing occupational function is. In terms of the treatments examined, IPSRT consisted of more sessions over a longer period of time than either study. In addition, compared with the functional remediation program used by Torrent et al., IPSRT did not have focus on addressing neurocognitive issues, and was conducted in individual sessions which were necessarily more focused on the problems presented by each individual. Rosa et al. (24) found a significant improvement in all 6 domains of the FAST scale over 6 months of intervention. The global improvements in functioning may be due to the hospital-based multi-disciplinary nature of the program, which had greater contact time during the study period than our own trials.

One review of disability in BD found only 5 of 34 studies examined assessed occupational outcomes, suggesting limited effort in addressing occupational problems associated with BD (38). Frank et al. (19) showed an initial improvement in occupational functioning on the UCLA Social Attainment Scale, when comparing patients assigned to acute IPSRT with acute ICM. We did not find a significant improvement of occupational outcomes measured by the work SAS-SR subscale. This difference may relate to stage of illness given Frank et al. examined patients who were in episode at baseline. In our studies, 68% of patients in study 1 were in episode while in study 2 none were in episode. Early gains in occupational functioning may be more likely when people are unwell at baseline, particularly as patients who are unemployed may be able to seek employment after early remission of mood symptoms and functional impairment as a result of psychotherapy. In our study, 19 patients gained employment over the course of 18 months and had work SAS-SR subscale scores recorded at week 78. However, they were unable to be included in analyses of the work subscale due to the lack of a baseline score. This clearly represents significant improvement which was not, however, measured using the SAS-SR. Conversely, 17 patients lost employment over the course of 18 months and did not have work SAS-SR subscale scores recorded at week 78, and were therefore excluded from analyses. This likely represents deterioration in patients' ability to work, but is unable to be interpreted by the SAS-SR. There were no significant differences between treatment randomizations for number of work gainers or losers.

Our results indicate that BD patients undergoing IPSRT as opposed to TAU achieved improved functional outcomes of social and leisure activities and extended family relationships. Similarly, patients undergoing IPSRT demonstrated significant improvements over time in the housework and social and leisure activities subscales. As many BD patients prioritize functional outcomes over symptomatic recovery (6), identifying patients who exhibit significant impairment in these domains may be beneficial in implementing appropriate therapies for patient-prioritized outcomes. We did not demonstrate any significant improvements in the work, marital, extended family, children or family SAS-SR subscale scores after 18 months treatment with IPSRT. This result may indicate the lack of global applicability for functional assessments in BD, as the number of respondents for each of these subscales dropped dramatically. As the topic of functional assessment and functional improvement gains traction in psychotherapeutic research, our results suggest that a closer examination of domain-specific outcomes is warranted to accurately understand improvements in function. Clarification of the concept of functioning in BD, and refinement of measures, is therefore needed (39).

This secondary analysis of two RCTs for BD has several limitations. Firstly, it should be noted that the studies were not designed to be pooled. Each had different inclusion and exclusion criteria and primary outcome measures. Patients were younger, more unwell at baseline and had poorer functioning in study 1. We adjusted for this by covarying in our analyses for variables that were significantly different between treatment arms at baseline. However, this may not correct for unmeasured differences between the samples. In addition the determinants of functional impairment may be important mediators of change and while we measured and examined several clinical variables, we did not, for example, examine stage of illness. Staging models have suggested progressive functional impairment in some individuals with BD (40). Future studies assessing domain-specific effects of treatment could usefully incorporate staging into their design.

Secondly, our analyses was limited by the number of patients that dropped out over the 18 month period, particularly in the TAU group. Patients in this study were relatively well at baseline, and TAU patients had less interaction with the study team which may account for this discrepancy. We compared baseline characteristics between dropout groups and found significant differences between treatment arms only on characteristics which were identified as likely to be different based on the different inclusion criteria of the studies analyzed (age, age of onset of any affective episode, and current episode). Thirdly, the age range captured in our studies was narrow with patients predominantly aged between 20 and 40 years, despite the lack of age restriction in study 2. This may contribute to the lower number of respondents in the work, marital, children and family unit subscales. Fourthly, the loss of data from particular subscales of course reduces power in the analysis of these subscales and potentially explains the lack of statistical improvement demonstrated in these domains of functioning and the lack of difference between IPSRT and TAU in the same domains (see Table 5).

In summary, in this *post-hoc* combined analysis of 152 BD patients undergoing 18 months of psychotherapy, there was a significant effect of IPSRT compared with TAU on the SAS-SR social and leisure activities subscale and extended family subscale, and a significant effect of IPSRT over 18 months on the SAS-SR housework and social and leisure activities subscale. This finding has similarities and divergences from previous functional domain-based studies. There are few studies that have assessed function in BD patients from a domain perspective, limiting understanding of patient function and improvement as a result of psychotherapy. We recommend that studies assessing patient function need to reflect the domains identified as relevant rather than those identified by clinicians.

## Data Availability Statement

The raw data supporting the conclusions of this article will be made available by the authors, without undue reservation.

## Ethics Statement

Ethical review and approval was not required for the study on human participants in accordance with the local legislation and institutional requirements. Written informed consent to participate in this study was provided by the participants' legal guardian/next of kin.

## Author Contributions

WM analyzed the data and wrote the first draft. RP supervised analysis and writing. CF supervised analysis. MC, MI, and KE were involved in planning of the analysis. All authors contributed to subsequent drafts. All authors contributed to the article and approved the submitted version.

## Funding

RP had use of computer software at no cost for research—provided by SBT-pro. Received support for travel to educational meetings from Servier and Lundbeck.

## Conflict of Interest

The authors declare that the research was conducted in the absence of any commercial or financial relationships that could be construed as a potential conflict of interest.

## Publisher's Note

All claims expressed in this article are solely those of the authors and do not necessarily represent those of their affiliated organizations, or those of the publisher, the editors and the reviewers. Any product that may be evaluated in this article, or claim that may be made by its manufacturer, is not guaranteed or endorsed by the publisher.
